# Older persons’ experiences with wearable sensor-based fall risk screening in free-living conditions - a qualitative study

**DOI:** 10.1186/s12877-025-06100-7

**Published:** 2025-06-21

**Authors:** Madelene Törnblom, Kari Rönkkö, Kerstin Ådahl, Staffan Karlsson, Ulrika Olsson Möller, Anna Nivestam

**Affiliations:** 1 Faculty of Health Sciences, Kristianstad University, Kristianstad, SE-291 88 Sweden; 2 Faculty of Business, Kristianstad University, Kristianstad, Sweden; 3 School of Health and Welfare, Halmstad University, Halmstad, Sweden; 4 Department of Health Sciences, Lund University, Lund, Sweden; 5 Institute for Palliative Care, Lund University and Region Skåne, Lund, Sweden

**Keywords:** Aged, Balance, Community-dwelling, Fall risk assessment, Falls, Interviews, Person-centredness, Physical activity, Preventive home visits, Sensor, Technology

## Abstract

**Background:**

Falls are common among older persons and can have a major impact on their lives. Wearable sensors used in free-living conditions (moving naturally in one’s daily living environment) can be used to predict falls and fall risks. To understand if using the wearable sensors is an acceptable way for older persons to be screened for fall risks, it is important to have knowledge of older persons’ experiences using wearable sensor-based technologies for fall risk assessment in free-living conditions Therefore, this study aimed to describe older persons’ experiences of using such technology.

**Methods:**

A qualitative study using individual interviews was conducted with 21 community-dwelling older persons (aged 77–81) in Sweden between April and September 2024. The older persons wore a thigh-mounted wearable sensor for one week to screen for fall risks in free-living conditions. Interviews were conducted 9–89 days (median 15 days) after sensor use and were analysed using conventional qualitative content analysis.

**Results:**

Older persons’ experiences with wearable sensor-based fall risk screening were described using the overarching theme ‘Being an older person in a fall screening process’ containing five categories: ‘Seeing a need for a fall risk sensor but imagining it as an unattainable ideal’, ‘Utilising a wearable sensor can be uncomplicated and fun’, ‘Having worries and experiencing problems’, ‘Thinking about what the wearable sensor has registered about me’, and ‘Reflecting on how I can benefit from the screening’.

**Conclusions:**

The older persons had various experiences with the wearable sensor-based screening for fall risks in free-living conditions. The wearable sensor was easy to use, although problems could occur while wearing it, such as losing the sensor or developing skin problems. The older persons wanted to benefit from the screening and improve their health based on the results. Further research could focus on the accuracy of fall predictors used in free-living conditions for assessing fall risks in older persons, since the wearable sensor was perceived as acceptable to use.

**Supplementary Information:**

The online version contains supplementary material available at 10.1186/s12877-025-06100-7.

## Background

Falls are common among older persons and can have a major impact on a person’s life. Each year, around 30% of all persons aged 65 years and older experience a fall [1, 2], and the number increases by age to 50% for persons over the age of 80 [3]. Balance disorder, fear of falling, history of falls, vision impairments, depression, age, dementia, and female gender are significant risk factors for falls in older persons [4]. For older community-dwelling persons, most falls occur in their homes or their close surroundings [5]. Injuries reported after a fall range from superficial cuts, bruises, and sprains to fractures and death [2]. The consequences of a fall can be reduced activity, fear of falling, decreased quality of life, less independence, and reduced confidence [5]. Current guidelines for fall prevention recommend that older persons at high risk of falling should be offered comprehensive multifactorial fall risk assessment and personalised interventions [6]. The multifactorial fall risk assessment should include mobility, muscle strength, medications, medical and cognitive conditions, sensory function, nutrition, environment, and psychosocial factors [6]. Exercises that target balance, gait, and muscle strength can be used to prevent falls [6–8], but personalised interventions can also include education about fall risks and environmental modifications [6]. Assessing a person’s movement patterns is therefore important, both to provide information that can personalise the exercise intervention and to develop adequate and acceptable methods to identify persons, especially older persons, at high risk of falling.

Various methods have been used to identify older persons at risk of falling. Commonly, both self-reported and performance measures for fall risk assessment are used [9]. Subjective measures (i.e. self-reported), such as question-based tools, can be used by either older persons or healthcare personnel. In a study by Frisendahl et al. [10], older persons found the use of a question-based screening tool for fall risk in clinical settings to be meaningful, as it increased their awareness of their own abilities. The older persons felt motivated to prevent falls, but a low fall risk could create a false sense of security. However, the predictive value of question-based tools for fall risk assessment has been questioned and it is recommended that healthcare personnel should use the tools with caution due to the tools’ limitations in identifying persons at risk [11]. Alternative methods to measure fall risk include objective measures (i.e. performance), including physical tests of gait and balance, and functional mobility assessment [12]. However, the predictive ability of the most used objective measures for fall risk assessment in community-dwelling older persons is inconsistent [12]. This is because no single physical test to assess objective measures can be used alone to predict falls in community-dwelling older persons [12]. These findings point to the need for alternative methods for assessing fall risks among older persons more accurately.

One method that could be used to identify older persons at risk of falling is wearable sensor-based technologies for objective fall risk assessment. Although there is emerging evidence that wearable sensors could be effective for preventing falls, current guidelines for fall prevention do not support the use of wearable sensors, as this evidence is considered inadequate [6], underscoring the need for further research. Research on wearable sensor-based technologies for fall risk assessment in older persons has increased over the last decade [13]. With the use of sensing technology, a new opportunity to measure fall risks in older persons has emerged [14], which is to measure a person’s way of moving around in free-living conditions. This is one of several assessments that are important in a comprehensive fall risk assessment. Free-living is when a person is in their natural daily living environment [15], moving around as usual. The use of wearable sensor-based technologies could investigate free-living fall predictors, such as time spent walking, time spent lying, step time, and number of turns, all of which may have the potential to be used to assess fall risks [15]. Wearable sensors used in free-living conditions have the potential to record more comprehensive data in daily life than physical tests, potentially improving fall prediction accuracy. Sensor technology has been shown to be a viable tool for risk assessment [14]. However, the older persons’ experience of using sensor technology needs to be further investigated [14]. This highlights the importance of studies that examine older persons’ experiences with wearable sensor equipment to assess whether it is user-friendly and whether they accept it as a method for fall risk screening.

To our knowledge, there is limited research examining older persons’ experiences using wearable sensor technologies to assess fall risk related to human movement in free-living conditions. Recent research has shown that age, gender, education, perceived usefulness, and perceived ease of use are some of the factors influencing older persons’ attitudes towards the adoption and use of technology [16]. For older persons, it is important that technology is need-driven and makes them feel comfortable [16]. Regarding wearable sensors to measure activity, a study by Reeder et al. [17] involving interviews with older women about wearable and smart home sensors measuring activity, revealed that sensor preference was largely influenced by lifestyle. Women with more active lifestyles found wearable sensors particularly useful. The study, conducted in a laboratory setting, reported that the women considered the use of wearable sensors for personal activity data collection to be acceptable [17]. Moreover, Moore et al. [18] reported that older persons are more likely to adopt wearable devices when they perceive a clear benefit to doing so. The research shows that older persons are less likely to use technology that might limit their independence [18]. More research is needed to better understand older persons’ experiences regarding the use of wearable sensor-based technologies for fall risk assessment using free-living fall predictors. Therefore, the aim of this study was to describe older persons’ experiences with wearable sensor-based fall risk screening in free-living conditions.

## Methods

### Design

The study had a qualitative design in which individual interviews were used [19] to describe older persons’ experiences with wearable sensor-based fall risk screening. In the design and report of the study, the Consolidated Criteria for Reporting Qualitative Research (COREQ) was used [20].

### Context

**Fig. 1:**
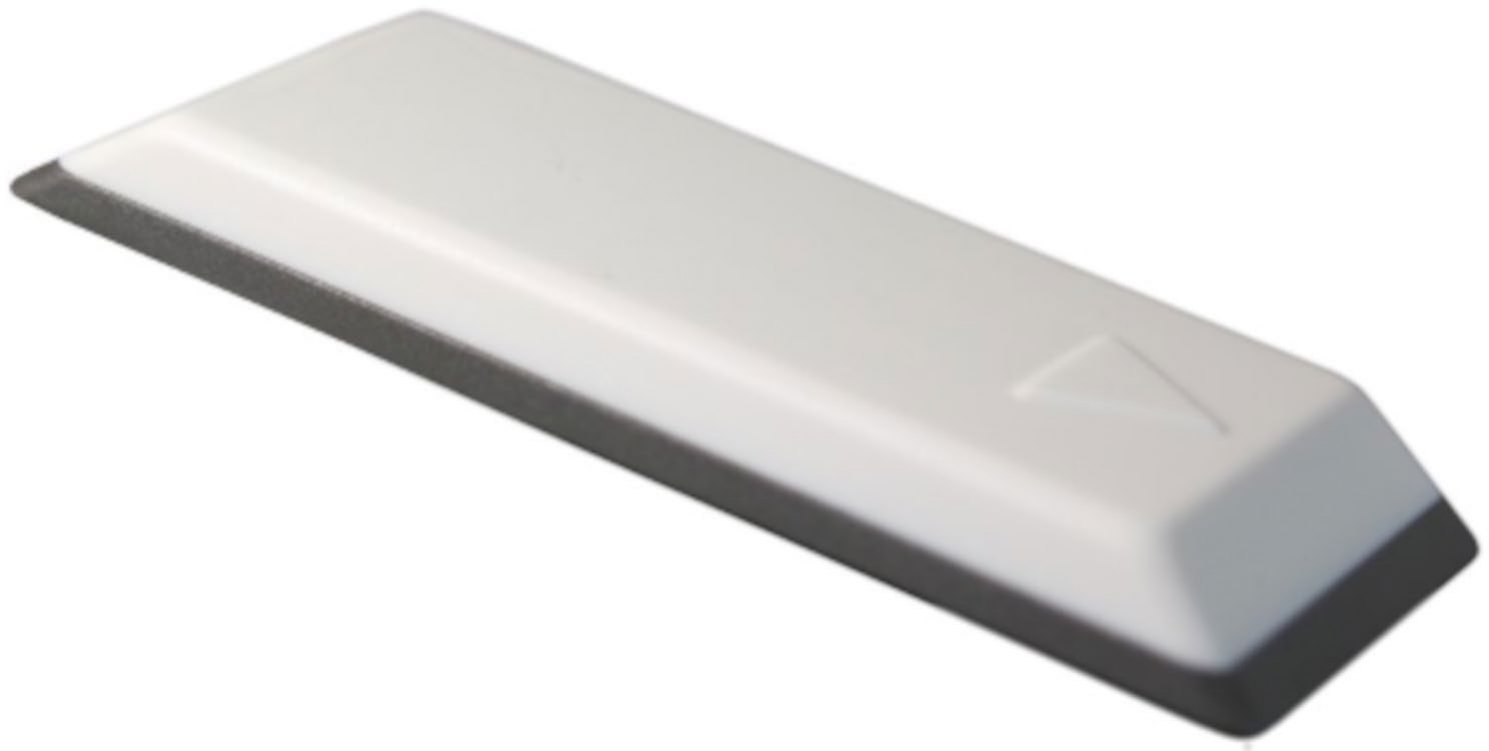
The wearable sensor, Snubblometer® [23] (with permission from J. Källmén, Infonomy AB, Lund, Sweden)

This study was conducted in three municipalities in southern Sweden within the research and collaboration project Preventive Home Visits to Seniors (Pre-H) [21, 22]. In brief, the purposes of the home visits are to give person-centred recommendations to community-dwelling older persons aged around 77 and without home care, to promote health, well-being, and independence, and to collect data on the older person’s situation for community planning. During a dialogue, the health personnel asked the older person questions. The questions were about, for example, the older persons’ health, and living situations. The answers were registered in a digital support system. Participants who had been purposively selected and offered a preventive home visit in Pre-H were invited to test wearable sensor-based screening in a research project named ‘Sensor-based fall risk screening in preventive home visits for older persons’ (the PreSens project). The present study is one of the studies conducted within the project. In the PreSens project, a wearable sensor named the “Snubblometer^®^” (size 7.9 × 3.2 × 0.8 cm) was being evaluated for fall risk screening in older persons (Fig. 1). The wearable sensor is an inertial measurement unit conducting measurements using three-axis gyroscopes and accelerometers that collect position and movement data at 50 Hz. The information is visualised in graphical interfaces. Variables measured by the Snubblometer^®^ are the number of steps, time spent standing/walking/resting (min), step length (cm), stability from 0-100 (unitless), step duration (ms), cadence (ps), number of possible fall events when the wearable sensor is set to sensitive, gait speed (m/s), number of times standing up, and angular velocity 20–70 degrees (degrees/s). These measures will be used in future studies within the PreSens project to investigate whether this wearable sensor can predict falls.

The health personnel in Pre-H informed the older person about the wearable sensor during a preventive home visit, and older persons interested in using the wearable sensor were then contacted by the first author by phone. The wearable sensor was delivered by the first author to the older person’s home. Either the first author or the older person attached the wearable sensor to the outside of the older person’s thigh with adhesive. The older persons were instructed to “live as usual” while wearing the sensor for a week. The older persons were informed that they should contact the first author by telephone or e-mail if they experienced any problems during the screening, had questions about the wearable sensor or wanted to end their participation. After one week, the older person posted the wearable sensor back to the first author, and then the data was extracted from the wearable sensor.

As a part of the PreSens project, data from the wearable sensor was presented to the older person after the interview using graphs and bar charts on paper. The participants were also encouraged to fill in a fall journal for 12 months from the first day of wearing the sensor and return it every three months. Since the PreSens is an ongoing project investigating whether the wearable sensor can predict falls, this data is not included in the present study.

### Participants

To select participants for this interview study, purposeful sampling [19] was used. We strived to achieve a variation of experiences in the interview material. Therefore, older persons who had used the wearable sensor in the PreSens-project in February-August 2024 were selected based on variations in age, sex, living in an urban or a rural area, and fall risk according to the Downton Fall Risk Index. The first author selected participants eligible for the study to achieve variation in these items. In total, 26 presumptive participants were asked to participate in an interview when the wearable sensor was delivered to their home. Of these 26, five declined to be interviewed. Three participants declined due to a lack of time and two because their partner was interviewed in this study. The interviews continued until participants’ information was repeatedly confirmed across multiple sessions. The characteristics of the 21 participants included in the study are summarised in Table 1.

**Table 1:** Sample characteristics

Characteristics	*n* = 21
Age median (range)	81 (77–81)
Woman *n* (%)	13(62%)
Man *n* (%)	8 (38%)
Living in an urban area *n* (%)	15(71%)
Living in a rural area *n* (%)	6 (29%)
Fall risk according to Downton Fall Risk Index^a^*n* (%)	13 (62%)
No fall risk according to Downton Fall Risk Index^a^*n* (%)	8 (38%)

^a^ Self-reported responses from The Downton Fall Risk Index [24]

### Data collection and procedure

Before the interview, both verbal and written information was given, and the older persons signed a written consent to participate. Sample characteristics were collected by the first author when delivering the wearable sensor to the older person’s home. Individual interviews were conducted in the older persons’ homes. An interview guide with open-ended semi-structured questions was developed and then tested in three pilot interviews. The pilot interviews were conducted with two men and one woman aged 77–81 years, between 13 and 20 days after they had used the wearable sensor. The first and last authors listened to and discussed the audio-recorded pilot interviews. A few changes were made to the interview guide; the order of the questions was changed, three questions were removed since they did not suit the aim, and the wording of questions was changed to make them more understandable. After discussion among all the authors, the pilot interviews were considered methodologically sound, warranting their inclusion in the analysis. The final interview guide (Additional file 1) consisted of questions about: experience of using the wearable sensor, thoughts about the wearable sensor, and the possibility of getting advice based on data from the wearable sensor. Examples of questions were “Tell me about what it has been like to use the wearable sensor during the week.” and “What do you think about being able to get personal advice on physical activity, sedentary behaviour, and balance based on what the wearable sensor has registered about you?”. Follow-up questions were asked such as: “What kind of advice would you like to get?”, “Can you give an example?”, “Did the Snubblometer^®^ fall off at any point? and How did you notice that the wearable sensor was gone?” The interviews were conducted by the first author 9–89 days (median 15 days) after the person had used the wearable sensor. Most participants were interviewed after 9–28 days. Two participants were interviewed after 58 and 89 days, respectively due to sickness. A total of 21 interviews were conducted between April and September 2024. In six of the interviews, the older person’s partner (*n* = 5) or relative (*n* = 1) was present in the same room when the interview took place but did not take part in the interview. The interviews lasted between 15 and 40 min, with a median of 24 min. All interviews were audio-recorded and thereafter transcribed verbatim using Amberscript.

### Data analysis

The transcribed text was analysed according to conventional qualitative content analysis as described by Hsieh and Shannon [25]. The first author initially listened to the interviews several times and read through the transcribed text repeatedly to get an overall view of the material. The first author then identified all text parts that were relevant to the aim to describe older persons’ experiences of fall risk screening using wearable sensors in free-living conditions. The focus was then on analysing these parts. The first and last authors separately identified codes related to the aim of the study in the text in one interview and then compared and discussed their codes. The codes identified were similar, which made the first author confident in continuing to code the rest of the interviews. After this, the first author continued to identify meaning units and gave them a code. This was done in all interviews using NVivo 15. Examples of codes were: “No problems wearing the sensor”, “Active as usual”, and “Interesting with a follow-up”. The first author then sorted the codes, based on the same content, into meaningful clusters. From the meaningful clusters, the first author started to identify categories. The first and last authors discussed the clusters and categories continuously during the analysis. The process involved constant movement between the parts and the whole, between the transcribed texts, meaning units, codes, clusters, and categories. The first and last authors repeatedly discussed and adjusted the categories. Then the second author read a transcript from one interview and compared it with the written results of this study, and was thereby able to verify the results. The first author came up with an overarching theme representing all five categories. All authors discussed and adjusted the theme until a consensus was achieved. Finally, older persons’ experiences with wearable sensor-based fall risk screening in free-living conditions were described using an overarching theme and five categories. Citations from the interviews have been used when presenting the results to increase trustworthiness. All authors agreed upon the final results.

### Rigour

The rigour of this study was ensured through the application of the following criteria: credibility, transferability, dependability, and confirmability. These criteria aimed to minimise the risk of bias and enhance the accuracy and trustworthiness of the research results [26]. Pilot interviews were conducted to strengthen credibility, and the interviewer (the first author) tried to maintain an objective and open-minded stance to avoid influencing the data collection. Two researchers were involved in the coding process while the remaining authors, who had extensive experience in qualitative methodology, contributed to the analysis, further supporting the study´s credibility. To increase dependability, the analysis process has been described in detail. The research team engaged in ongoing reflexive dialogue throughout both the data collection and analysis to further enhance credibility. Any disagreements were resolved through consensus. Quotes from the participants were included to support the findings and to strengthen confirmability. Additionally, the participants, research context, and study procedures have been thoroughly described to enhance transferability.

### Ethics approval and consent to participate

The study was conducted according to the Declaration of Helsinki. The study was approved by the Swedish Ethical Review Authority, reference number 2023-03425-01. Before the interview, both verbal and written information were given to the older persons, and they gave their written consent to participate. If the older persons had problems related to the wearable sensor during the screening, they were encouraged to contact the researcher.

## Results

**Fig. 2:**
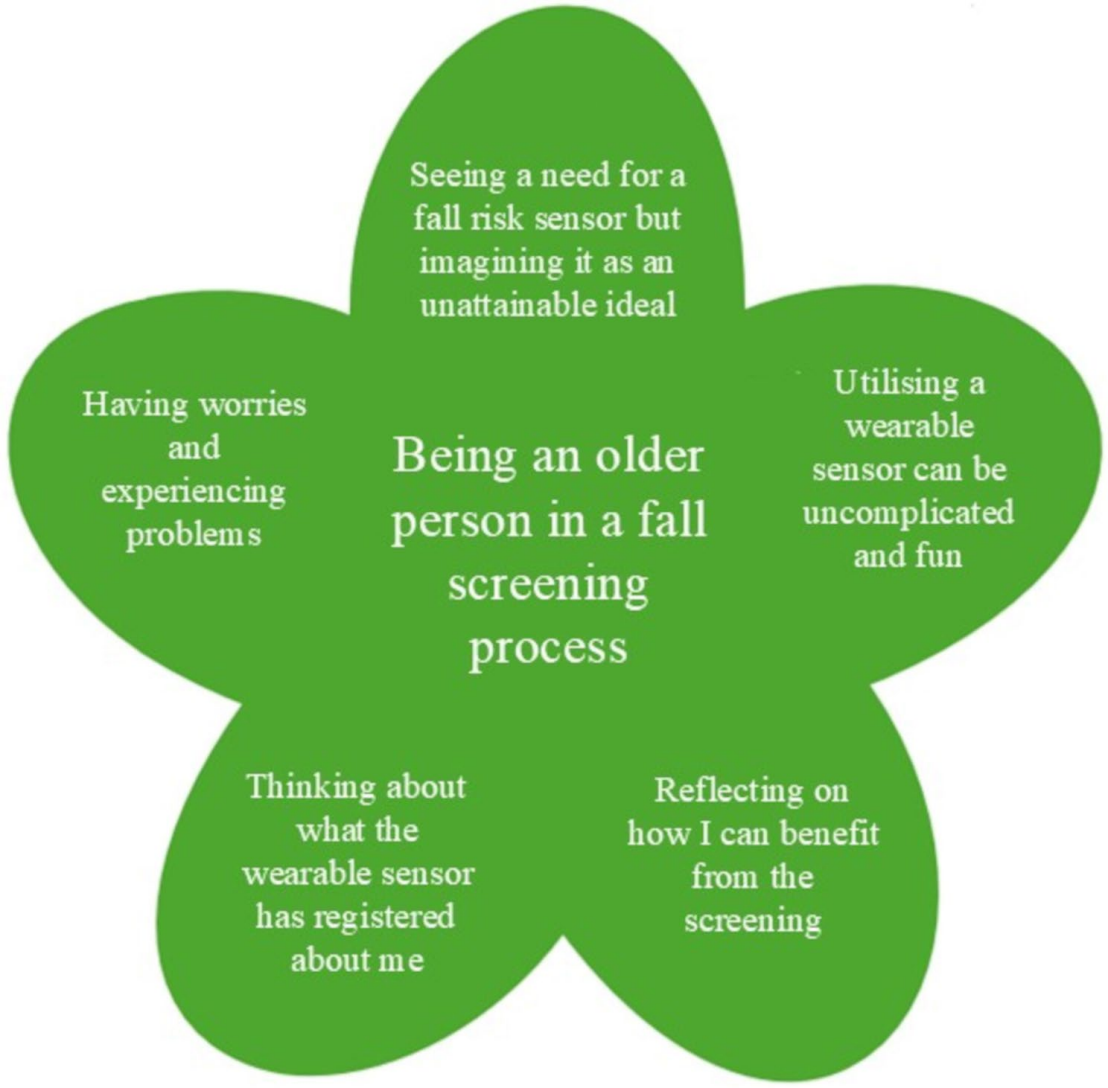
The overarching theme and five categories representing older persons’ experiences with wearable sensor-based fall risk screening in free-living conditions

Older persons’ experiences with wearable sensor-based fall risk screening were described using the overarching theme of *Being an older person in a fall screening process* and five categories: *Seeing a need for a fall risk sensor but imagining it as an unattainable ideal*, *Utilising a wearable sensor can be uncomplicated and fun*, *Having worries and experiencing problems*, *Thinking about what the wearable sensor has registered about me*, and *Reflecting on how I can benefit from the screening* (Fig. 2). Each person had unique experiences during the screening, highlighting the diversity of the participants’ perspectives. Despite the standardised nature of the screening, older persons experienced it in varied ways. They acknowledged the need for a fall risk sensor but viewed its purpose as somewhat idealistic. While utilising a wearable sensor could be straightforward and enjoyable, they also expressed concerns and encountered difficulties during the process. As persons with distinct thoughts and values, they wondered about what was being recorded about them and reflected on how they could benefit from the screening. Their experiences were influenced both by their personal thoughts and reflections and by uncontrollable events, such as losing the wearable sensor, during the process. Ultimately, they were all navigating the experience of being an older person in a fall screening process.

### Seeing a need for a fall risk sensor but imagining it as an unattainable ideal

This category demonstrates that older persons who felt at risk of falling said that they recognised the need for a fall risk sensor but perceived the wearable sensor’s predictive capabilities as an unattainable ideal. They felt the timing was right for wearable sensor-based screening, as they had recently experienced problems with balance, and their feet and legs. Some said that they had experienced a fall in recent years and had noticed a deterioration in balance.

In the interviews, the older persons expressed a desire to measure fall risks. They felt it was normal to have a higher fall risk at their age but wanted to know more about when and why the risk increases. They described a need for a fall risk sensor and said it would be good if this wearable sensor could measure fall risks. Undergoing fall risk screening with a wearable sensor made the older persons reflect on the various reasons why people fall, and they said they viewed fall risk prediction with the help of this wearable sensor as an unattainable goal since falling is influenced by many factors, some of which possibly could not be identified by the wearable sensor. For example, some said that a fall could occur if they took a risk or were careless. A fall due to carelessness was described as something that the wearable sensor could not predict.

*I’ve fallen. But it’s not because I suffer any imbalance*, *it was just carelessness/…/ how can it [the wearable sensor] know that I’m at risk or not huh /…/ A little word*, *what is it called u. utopia.* Person 6.

### Utilising a wearable sensor can be uncomplicated and fun

Utilising the wearable sensor was described as both uncomplicated and fun by the older persons. They appreciated the opportunity to use the wearable sensor and were positive about wearing it. Measuring one’s physical activity was described as exciting and fun. The older persons expressed concerns about handling and wearing the sensor but found the experience to be better than they had anticipated.

They felt that the application of the wearable sensor to the thigh was straightforward, both when they attached the wearable sensor to the thigh themselves and when they were assisted by another person. Applying the wearable sensor was straightforward as it was marked with an arrow showing the direction of placement on the thigh. They were afraid that it would be difficult to remove the wearable sensor from the thigh, but they found it easy when they tried. The skin looked normal after wearing the sensor, but the older persons said that the sensor was more difficult to remove if the adhesives were changed during the week.

The older persons reported that wearing the sensor did not interfere with their daily activities. They expected to feel the wearable sensor on their thigh, but in fact, they did not feel it and forgot they were wearing it. To become aware of the wearable sensor they needed to feel it with their hand. Only when they accidentally put their hand over the wearable sensor or knocked it did they remember that they were wearing it.

*No*, *no. It sat there so well /.../ It didn’t bother me. Not even when I was lying down. /.../ No. So there were no problems. /.../I didn’t think about having it*, *I just did what I usually do.* Person 12.

### Having worries and experiencing problems

The older persons felt responsible for the wearable sensor and the data collection, and they had some worries and experienced problems while wearing the sensor. They said that they were worried that the wearable sensor would fall off, and this made them change the adhesives once or several times. They felt that the wearable sensor was in the way when they slept on the side it was worn, and they said it woke them up. This made them change their sleep patterns at night. In the interviews, they said they were careful not to damage the wearable sensor or drop it, and refrained from certain activities, such as showering and exercising, as a result. When they were showering and during toilet visits, they said they were afraid that the wearable sensor would be accidentally torn off. In addition, they were careful and tried to lift their pants/underwear over it. They also said that they did not hold grocery bags against their thigh when shopping.

Other issues experienced included skin problems and itching while wearing the wearable sensor, in addition to redness and marks on the skin when the sensor was removed. An older person noticed a wound on the skin when the wearable sensor was removed, and this wound healed after a few weeks. This was experienced as worrying since the older person had recently had another difficult-to-heal wound.

The older persons said that the wearable sensor fell off one or several times. They explained that the wearable sensor could fall off by getting caught on clothes or could fall off spontaneously during a walk in the woods or while gardening. Losing the wearable sensor was described as a shock, especially if it was not obvious when or where it disappeared. Moreover, losing the wearable sensor was experienced as stressful, making it difficult to relax with the wearable sensor on, and sending back the wearable sensor was a relief. One older person lost the wearable sensor in the forest and was upset when it disappeared, and happy when it was found again. The older persons said it was good to discover that the wearable sensor could fall off. One older person put the wearable sensor back upside down as they had forgotten how it should be placed but then figured this out and attached it correctly again. When the wearable sensor fell off, the older persons wondered why it had happened, if there could be something wrong with the adhesive, or if there was some other reason why it had fallen off.

*I couldn’t relax and kind of forget about it; [the wearable sensor] was there in the back of my mind the whole time. So it was definitely a bit upsetting when it [the wearable sensor] disappeared. /…/ But thankfully I found it [the wearable sensor] again so I didn’t think about it anymore. There’s nothing else that stressed me out except that [wearing the sensor].* Person 9.

### Thinking about what the wearable sensor has registered about me

The older persons said they had thoughts about what had been registered about them. Being curious about the results from the screening, they wondered what the wearable sensor could reveal about them. They said that it felt okay that the wearable sensor collected data and also stated that they had no secrets. Some said they felt sorry if the wearable sensor had not registered anything about them. In the interviews, the older persons reflected that the data collected could reveal aspects of their daily life and routines they had not previously considered, for example, when they took breaks or rested during the day.

*That’s the reality. /…/ Sometimes it happens that I have the TV on while I’m cooking and then I might walk a little. But then*, *I hear something special [from the TV]… then I go in and sit in the armchair [for] maybe two minutes. /…/ These are all the pauses that you don’t think about but are there. The [wearable sensor] registers [in] a different way than what you register yourself. /…/* Person 3.

Moreover, the older persons found it interesting and exciting that the wearable sensor measured their activity. They said that it would be fun to see if they had moved enough. It was perceived as good if the wearable sensor could measure missteps and balance capacity because balance capacity deteriorates over the years. Uncertainties were expressed about what the wearable sensor had registered, for example, whether the wearable sensor could measure missteps and cycling. The older persons described how the results might vary if the measurement were conducted in a different week for example, if they had spent more time sitting in one week compared to another.

During the week they wore the sensor, the older persons said they acted as usual, doing their normal activities. However, they also said that to obtain comprehensive screening results, they increased their activity levels during the first few days, paying more attention to their movement patterns. Some described having an unusual week and they were not active as usual because of this, for example, having pain or other commitments they did not typically have. While wearing the sensor, the older persons kept a diary in which they noted anything special that happened. They emphasised that they had not fallen during the week they used the wearable sensor.

### Reflecting on how I can benefit from the screening

The older persons reflected on how they could benefit from the screening. To benefit from the results of the wearable sensor-based screening the older persons preferred the screening results to be presented on paper at a personal meeting or sent to them in an e-mail. They said that they would save the results from the screening, as it would be fun to be able to look at them afterwards. They could be compared, for example, with the pedometer readings on the mobile phone. The older persons felt that through the results they would find out new things about themselves. This information was reportedly used to benefit from the screening as it helped them to make changes to improve their health, such as taking more walks or starting exercise. On the other hand, some had continued as usual regardless of the screening results. This was because they felt it was natural to have poorer balance capacity, or they said they were already exercising enough. The screening results were described to be beneficial during healthcare meetings since the older persons could show their results to the healthcare personnel. The older persons said that receiving the results could increase their likelihood of seeking help. They also expressed a desire to use the wearable sensor again to benefit further. They thought it would have been interesting to redo the screening and capture changes over time.

If the screening showed an increased fall risk, the older persons reflected on what changes they would make to reduce the risk. For example, they highlighted that they had made adjustments in their homes. The older persons in this study said they might be more cautious if they were aware of their fall risk. They said they would think twice before doing challenging activities, pay closer attention to uneven ground while walking, or start using walking aids since they did not want to experience a fall. However, some stated they would maintain their usual activity levels despite knowing about increased risks.

The older persons highlighted that to benefit fully from the screening, they would like advice based on the data collected with the wearable sensor. They pointed out that technology could not give advice. They said that others knew more when it came to giving advice and that they trusted health personnel to do this. Personal advice would be appreciated, and it would have been beneficial to know if they needed to change anything.

*I think it’s great [that the wearable sensor measured activity]*, *because you don’t usually think about that much. And you can come to realise that you’ve moved too little. That’s very good because then you can start moving a little more. /…/ [if I had a risk of falling] then I would probably have thought that I had to be a little more careful. Yes.* Person 16.

However, although receiving advice was viewed positively, the older persons mentioned being unwilling to follow it, physically unable to do so, or being uncertain whether the advice had actually helped them.

## Discussion

This study aimed to describe older persons’ experiences of fall risk screening using wearable sensors in free-living conditions. Although they all participated in the same screening process, the older persons in this study experienced it differently. Being different persons, undergoing the screening resulted in various descriptions of the experience. The older persons expressed a desire to measure fall risks and thought having poor balance was a natural part of ageing. They described it as easy to utilise the wearable sensor. However, some older persons experienced problems, such as a wound that took time to heal or difficulties sleeping on the side they wore the sensor. They also felt worried that they would lose the wearable sensor. The older persons reflected on what had been registered about them and wanted to be able to use the registered data and results from the screening to improve their health. However, some of them said they would not make any changes regardless of what the results might show. In summary, the results showed different experiences of being an older person in a screening process.

The older persons considered that the wearable sensors used to predict falls in free-living conditions were uncomplicated to handle and wear. The current study showed that the wearable sensors were easy to utilise. Since they found it easy to wear the sensor, they were eager to use it again. They also saw a benefit in getting the results from the screening and doing follow-ups over time, trying to make changes to improve their health. This is consistent with previous research showing that older persons’ attitudes towards using technology are affected by how easy it is to use [16] and that older persons are more likely to adopt wearable devices when they perceive a clear benefit [18]. However, the older persons in the present study also experienced some problems wearing the sensor. Some had trouble sleeping on the side they wore the sensor and woke up because of this at night. One older person suffered a wound caused by the wearable sensor that took time to heal and found this worrying, while some lost the wearable sensor and found this to be stressful. Older persons may have a negative attitude towards technology if it disrupts their lifestyle [16], and negative experiences of handling and wearing the sensor could therefore affect older persons’ attitudes towards using technology. Therefore, these problems need to be further investigated to determine how often they occur and how they can be handled, to reduce the inconvenience for older persons utilising wearable sensors like this.

Person-centred advice based on results from the wearable sensor could be given during health interventions. In this study, the older persons said that personal advice based on the data collected from the wearable sensor would be appreciated. They also said that they trusted health personnel to give advice. Frisendahl et al. [10] report that older persons who had a higher fall risk when being screened for fall risk described it as important to get support from health personnel to reduce their risk. One health intervention where preventive advice is given is preventive home visits to older persons, and fall prevention advice is among the most frequently given advice during preventive home visits [27]. Nivestam et al.’s [28] study showed that older persons receiving a preventive home visit relied on health personnel to assess their health and give advice. The older persons in that study said that the advice given during the home visit contributed to a feeling of becoming recognised as a person [28], indicating that the visits had a person-centred approach. Having a person-centred approach when addressing fall risks is recommended in current guidelines for fall prevention [6]. The guidelines also state that all older persons should be advised on fall prevention and physical activity [6]. Hence, preventive home visits seem to be a suitable arena for using wearable sensors like the ones described in this paper and for giving person-centred advice based on the data collected using the wearable sensor.

Being aware of an increased risk of falling and having concerns about it can limit the activities of older persons. In this research, the older persons said that they might be more careful if the wearable sensor showed that they had a risk of falling. For example, they would think more about the unevenness of the ground while walking or would start using a walking aid. Concerns about falling can cause limitations in usual activities and increased fall risk as a result [6]. In recent research by Frisendahl et al. [10], older persons said that a fear of falling could increase their fall risk. This could result in increased health risks for the older person. On the other hand, the older persons in this research also said that they would be as active as usual even if they knew the wearable sensor showed that they had an increased risk of falling. This emphasised the importance of viewing older persons as a person, adopting a person-centred approach, and avoiding a one-size-fits-all approach when addressing fall risks or providing fall prevention advice [6].

### Strengths and limitations of the study

To increase the trustworthiness the COREQ [20] checklist was used. The older persons in this study had all used the wearable sensor and had the experience needed to meet the aim of the study. The sample was selected purposefully to obtain a variance in age, sex, living in an urban or a rural area, and fall risk according to the Downton Fall Risk Index among the older persons. This gave a broad variety of participants, supporting the transferability of the results. The participants in this research all expressed a positive or neutral attitude towards data collection with the wearable sensor, and persons with a negative attitude probably chose not to participate in this research. This means we could have missed important experiences that these persons would have described. All participants in the study were community-dwelling older persons who had no home care. It is possible that the participants therefore represented active and independent older persons. This means we may have missed describing the valuable experiences of persons who are not as active, older persons receiving home care, or living in nursing homes.

Since the first author had only minor previous experience of conducting interviews, she was guided by the other, more experienced authors, which increased the trustworthiness. Three pilot interviews were conducted to ensure that the questions in the interview guide suited the aim of the study. These pilot interviews were included in the analysis, which may be a limitation. However, the pilot interviews were discussed among all authors and included since they were considered methodologically sound. For two older persons, the interview took place after 58 and 89 days, respectively, due to sickness. Due to the risk of recall bias, the interviews were compared to the others, and no differences were found. They were therefore also considered methodologically sound and included in the analysis. The interviews lasted between 15 and 40 min, with a median of 24 min. However, the aim was to describe older persons’ experiences, not to gain a deeper understanding of the phenomenon. In addition to this, we continued to interview until the information from the participants was repeated in multiple interviews. To increase credibility, citations have been used when presenting the results. Credibility is also increased if more authors read through and analyse the text [26], so the second author read through one interview verifying the results.

## Conclusions

Being an older person participating in a fall risk screening process highlighted the various experiences that can come with wearing a sensor for a week. The screening was generally well accepted, even though some difficulties, such as losing the sensor or getting skin problems, were encountered. Wearing the sensor prompted the older persons to reflect on fall risks, fall prediction, the data being recorded, and how they might improve their health after receiving information based on the data collected by the wearable sensor. It could be suggested that the wearable sensor-based screening for fall risks could be tested in health interventions to evaluate whether it can identify persons with increased fall risks and improve the health of older persons. The wearable sensor could be part of a comprehensive fall risk assessment and used in person-centre interventions to reduce fall risks. However, to ensure a person-centred approach, health personnel who distribute wearable sensors to older persons need to be aware of the different experiences older persons may have with wearable sensor-based fall risk screening. Health personnel must also be aware that fall risk advice given to improve health could have the opposite effect. Since the wearable sensor was perceived as acceptable to use, further research could focus on the accuracy of fall predictors used in free-living conditions for predicting fall risks in older persons. It is also important to explore health personnel’s perspectives on wearable sensor-based screening in order to give person-centred advice based on the screening results. The problems during the screening should be further investigated to reduce the inconvenience for older persons while handling and wearing sensors.

## Electronic supplementary material

Below is the link to the electronic supplementary material.

Additional file 1: Interview guide (The interview guide used in this research)

## Data Availability

The transcripts from the interviews analysed during the current study are handled confidentially. They are available from the corresponding author on reasonable request.

## Abbreviations

COREQ: Consolidated Criteria for Reporting Qualitative Research
Pre-H: Preventive Home visits to Seniors
PreSens: Sensor-based fall risk screening in preventive home visits for older persons
