# Supplementary material for: Older persons’ experiences with wearable sensor-based fall risk screening in free-living conditions - a qualitative study
Source: BMC Geriatr. 2025 Jun 21;25:426. doi: 10.1186/s12877-025-06100-7 (PMC12181887; doi:10.1186/s12877-025-06100-7)
Supplement: Supplementary file 1 — Additional file 1: Interview guide (The interview guide used in this research) [file 12877_2025_6100_MOESM1_ESM.docx]

**Interview guide**

1. Tell me about what it has been like to use the wearable sensor during the week.
   1. Was there anything you found difficult?
   2. Did you move around as you usually do during the week?/Did the sensor have any effect on the way you moved?
   3. What has it been like to wear the sensor?
   4. Did wearing the Snubblometer® bring up any emotional reactions?
   5. Did the Snubblometer® fall off at any point? How did you notice that the wearable sensor was gone?
2. What were your first thoughts when you saw the Snubblometer®?
3. What do you think about the idea that the Snubblometer® could measure your risk of falling?
4. How do you feel about the Snubblometer® measuring your activity?
5. What do you think about the fact that the Snubblometer® could measure your balance?
6. How would you like the results from the Snubblometer® to be presented to you?
   1. How would you use that kind of result?
7. What do you think about the Snubblometer® collecting data about you?
8. What are your thoughts on the idea that sensors like this could affect fear of falling or balance?
9. What do you think about being able to get personal advice on physical activity, sedentary behaviour, and balance based on what the wearable sensor has registered about you? What kind of advice would you like to get?
10. Do you have any thoughts on when, in addition to this context, you could use a Snubblometer®?
11. What made you want to take part in this research?
12. How has it been for you to be part of this research so far?
